# A rapid quality control test to foster the development of the sterile insect technique against *Anopheles arabiensis*

**DOI:** 10.1186/s12936-020-3125-z

**Published:** 2020-01-23

**Authors:** Nicole J. Culbert, Nanwintoum Séverin Bimbilé Somda, Maiga Hamidou, Dieudonné Diloma Soma, Silvana Caravantes, Thomas Wallner, Mamai Wadaka, Hanano Yamada, Jérémy Bouyer

**Affiliations:** 1 Insect Pest Control Laboratory, Joint FAO/IAEA Division of Nuclear Techniques in Food and Agriculture, Vienna, Austria; 2 Institute of Integrative Biology & the Centre for Genomic Research, University of Liverpool, Liverpool, Merseyside UK; 3 Institut de Recherche en Sciences de la Santé/Centre Muraz, Bobo-Dioulasso, Burkina Faso; 4 Laboratoire d’Entomologie Fondamentale et Appliquée (LEFA), Université Ouaga 1 Professeur Joseph Ki-Zerbo, Ouagadougou, Burkina Faso; 5 Université Nazi Boni de Bobo-Dioulasso, Bobo-Dioulasso, Burkina Faso

**Keywords:** Sterile insect technique (SIT), Malaria, Flight ability, Quality control (QC)

## Abstract

**Background:**

With the fight against malaria reportedly stalling there is an urgent demand for alternative and sustainable control measures. As the sterile insect technique (SIT) edges closer to becoming a viable complementary tool in mosquito control, it will be necessary to find standardized techniques of assessing male quality throughout the production system and post-irradiation handling. Flight ability is known to be a direct marker of insect quality. A new version of the reference International Atomic Energy Agency/Food and Agricultural Organization (IAEA/FAO) flight test device (FTD), modified to measure the flight ability and in turn quality of male *Anopheles arabiensis* within a 2-h period via a series of verification experiments is presented.

**Methods:**

*Anopheles arabiensis* juveniles were mass reared in a rack and tray system. 7500 male pupae were sexed under a stereomicroscope (2500 per treatment). Stress treatments included irradiation (with 50, 90, 120 or 160 Gy, using a Gammacell 220), chilling (at 0, 4, 8 and 10 °C) and compaction weight (5, 15, 25, and 50 g). Controls did not undergo any stress treatment. Three days post-emergence, adult males were subjected to either chilling or compaction (or were previously irradiated at pupal stage), after which two repeats (100 males) from each treatment and control group were placed in a FTD to measure flight ability. Additionally, one male was caged with 10 virgin females for 4 days to assess mating capacity (five repeats). Survival was monitored daily for a period of 15 days on remaining adults (two repeats).

**Results:**

Flight ability results accurately predicted male quality following irradiation, with the first significant difference occurring at an irradiation dose of 90 Gy, a result which was reflected in both survival and insemination rates. A weight of 5 g or more significantly reduced flight ability and insemination rate, with survival appearing less sensitive and not significantly impacted until a weight of 15 g was imposed. Flight ability was significantly reduced after treatments at 4 °C with the insemination rate more sensitive to chilling with survival again less sensitive (8 and 0 °C, respectively).

**Conclusions:**

The reported results conclude that the output of a short flight ability test, adapted from the previously tested *Aedes* FTD, is an accurate indicator of male mosquito quality and could be a useful tool for the development of the SIT against *An. arabiensis*.

## Background

Malaria is a disease that still accounts for almost half a million deaths each year. The latest report published by the World Health Organization (WHO) on malaria highlighted that although fewer deaths were reported in 2017, the success in global malaria control has stalled [1]. Such statistics underline the urgency for alternative complementary control measures. The sterile insect technique (SIT) is one of several genetic control measures routinely used throughout the world to suppress, contain or eradicate various species of agricultural, veterinary or human insect pests [2–4]. A technique which has proved successful and sustainable, particularly when deployed as part of an area-wide integrated pest management programme (AW-IPM).

Despite strong efforts in the 1970s and 1980s to roll out the technique against mosquitoes, one of the most devastating vectors of human disease, efforts were more or less abandoned until recent years. A build-up of insecticide resistance coupled with the global spread of species, such as *Aedes aegypti* and *Aedes albopictus,* has reignited interest in developing mosquito SIT as part of an AW-IPM approach, with the technique progressing towards implementation at an operational level. Mass-rearing technology has been standardized [5–7] and guidelines created for key vectors including *Anopheles arabiensis* [8], *Ae. aegypti* and *Ae. albopictus* [9]. More recently, research has arisen addressing some key elements of the handling, transport and release aspects of a mosquito SIT programme. Optimal transportation conditions have been advised for *An. arabiensis* [10] in addition to a study investigating various release conditions for both *An. arabiensis* and *Ae. aegypti* [11].

The ability of an insect to perform flight is known to be a direct marker of their quality [12]. Poor fliers will be unable to complete their primary goal which is to seek out and mate with wild females. Thus, it is imperative that in any SIT programme, insects released are of adequate quality. Quality control (QC) checks must be carried out routinely throughout critical steps of the mass-rearing process and both pre- and post-release. Flight cylinders that can gauge flight ability have consistently been reported to be an accurate indicator of mating competitiveness and are routinely used as a QC tool in fruit fly, tsetse fly and moth SIT programmes [13, 14]. Such cylinders offer an easy to use, portable and rapid means of estimating sterile insect quality both pre- and post-release.

Until recently, flight mills were the only method of assessing flight ability. Such tools require a high level of skill and the process of the flight test can be lengthy. The first mosquito QC tool was reported by Balestrino et al. [15] which measured the flight ability of recently emerged *Ae. albopictus* from pupae that had been placed in 1 of 100 individual wells. Despite promising results, the test itself required a period of 48 to 72 h to complete. However, the basis of this device was used to create a new flight cylinder that could measure the flight ability of 100 adult *Ae. aegypti* or *Ae. albopictus* mosquitoes within a 2-h period. A series of 10 prototypes were created and verified by a series of stress treatments such as irradiation, chilling temperature and compaction [16]. The final device is now considered as the IAEA reference QC test for *Aedes* mosquitoes.

In order to expand the applicability of this device to *An. arabiensis,* it was necessary to first modify the diameter of the individual flight tubes. Initial experiments in the *Aedes* device with male *An. arabiensis* were unsuccessful as their larger body size prevented them from completing vertical flight to escape the tubes. A new prototype FTD was constructed in exactly the same manner as with the first version for male *Aedes* mosquitoes with one simple adaptation; the diameter of each individual flight tube was increased from 8 to 10 mm. Preliminary experiments with the new prototype proved successful and thus a series of 10 new FTDs were constructed in order to validate the flight ability test as an indicator of overall quality via a series of stress treatment experiments as was done when validating the initial FTD for male *Ae. aegypti* and *Ae. albopictus.* Male *An. arabiensis* were exposed to a range of irradiation doses, chilling temperatures and levels of compaction and subsequent flight ability measure. To further link flight ability to quality, male survival and mating capacity were also investigated as reference tests (Additional files 1, 2, 3).

## Methods

### Mosquito colony source and mass rearing procedure

All experiments were undertaken using the Dongola strain of *Anopheles arabiensis* sourced from the Northern State of Sudan (Tropical Medicine Research Institute, Khartoum). The laboratory colony was maintained at the Insect Pest Control Laboratory (IPCL) of the joint Food and Agricultural Organisation/International Atomic Energy Agency (FAO/IAEA) Division of Nuclear Techniques and Agriculture, Seibersdorf, Austria since 2005. Larvae were mass-reared in plastic trays held in a mechanized stainless-steel rack developed at the IPCL [6]. Eggs were quantified using the method defined in Maiga et al. [7] wherein 4000 eggs are added to each tray, containing 4 l of deionized water, within a plastic ring which floats on the water surface. Water was added to each tray 24 h before the addition of the eggs to allow the temperature of the water to acclimatize to that of the ambient air temperature. Larvae were fed daily on a 1% (wt/vol) diet developed by the IAEA and described in [17], following the feeding regime described in the guidelines for mass rearing *Anopheles* mosquitoes [18]. Larvae were maintained in a large climate-controlled room (88 m^2^) with temperature and humidity maintained at 30 ± 1 °C, 70 ± 10% RH, respectively.

Adults were maintained under controlled temperature, RH and light regimes (27 ± 1 °C, 70 ± 10% RH, 12:12 h light:dark (L:D) photoperiod with 1 h periods of simulated dawn and dusk). Eggs were generated and gathered for all experiments following the *An. arabiensis* mass-rearing guidelines developed at the IPCL [8]. Mosquitoes were housed in standard plastic rearing cages (30 × 30 × 30 cm—Bugdorm, Taipei, Taiwan) and provided with constant access to a 5% sugar solution until day 3 when experiments were performed. Mosquito maintenance and the age of the adults at the time all described experiments were performed were chosen to reflect what may occur in a mass rearing facility prior to a release of sterile males. Despite *An. arabiensis* males reaching sexual maturity within 48 h of emergence, males younger than 3 days old display low insemination rates [19]. There were two replicates for each stress treatment in addition to two control samples for each experiment performed.

### Irradiation procedure and experimental design

On the first morning that pupation was noted (7 days after eggs were placed in the trays), the rack was tilted. Pupae and larvae were separated by adding them to an Erlenmayer flask containing dechlorinated water and swirling to create a vortex. The difference in buoyancy and swimming behaviour between the larvae and pupae, during which the pupae rise to the surface and the larvae swim downwards, allows an accurate separation of the two life stages [20]. The first observed pupae were discarded from the experiment and the larvae placed back into mass rearing trays. After a period of 2 h, pupae were then separated from the larvae once more ensure that the age of the experimental pupae was between 0 and 2 h. Pupae were handled using disposable transparent pipettes. Males were separated from females by distinguishing differences in their genitalia under a stereomicroscope while placed on a transparent petri dish lid [20]. Pupae were irradiated at 24 ± 2 h in batches of 250 inside a self-contained ^60^Co Gamma Cell 220 (Nordion Ltd, Kanata, Ontario, Canada). A dosimetry system using Gafchromic MD film was used to verify absorbed dose. A range of irradiation doses required to induce full sterility and beyond, and thus expected to reduce the quality of the adult males, were selected. Control males were taken to the Gamma Cell room but not placed inside or exposed to any irradiation dose whilst experimental males received a dose of 50, 90, 120 or 160 Gray (Gy).

### Chilling procedure and experimental design

Following on from previously reported research from IPCL [10], a range of chilling temperatures that were known to be tolerable (4, 8 and 10 °C), were selected, A lower temperature of 0 °C was chosen with the intention to reduce quality post-chilling as it was beyond the range of temperatures previously tested and found to be tolerable. On day 3, cages of 250 adult males were immobilized at each of the above temperatures for a period of 2 h, with control males left under insectary conditions (27 ± 1 °C).

### Compaction procedure and experimental design

On day 3, batches of 250 adult male *An. arabiensis* were immobilized at 10 °C for a period of 2 h. As noted above, 10 °C has been found not to impact quality. During immobilization, as described above, adult males were subject to varying levels of compaction by being placed under a mosquito substitute particle—cumin seeds. Cumin seeds were selected after analysing the morphological properties and weights of several substitute particles such as fennel, rice, anise and poppy seeds. Cumin seeds were found to best match the weight and characteristics of adult mosquitoes and were thus selected.5, 15, 25 or 50 g of cumin seeds were weighed, wrapped in mesh and sealed with an elastic band corresponding to 0, 0.25, 0.76, 1.27 and 2.55 g/cm^2^ respectively.

### Assessing survival rate and mating propensity as a measure of quality

The aim was to link post stress-treatment flight ability with survival rate and mating capacity which are known quality parameters. Firstly, survival rate was measured by removing and quantifying the number of dead adults in both experimental and control cages for 15 days post stress-treatment in both replicates. The number of adults available for the longevity experiment (N), after flight ability and mating capacity tests, ranged between 117 and 227 for the irradiation experiment. For the chilling and compaction experiments, N ranged between 106–149 and 77–112, respectively. Mating capacity was evaluated by placing a single control or experimental male from each treatment into a standard cage (30 × 30 × 30 cm) with 10 virgin females from the same cohort for a period of 5 days, with continued access to a 5% sugar solution. Females were then dissected and the spermatheca removed and viewed under a stereomicroscope for the presence or absence of sperm to clarify the insemination rate. Five repetitions were carried out for both the control and experimental treatments.

### Flight test device and experimental procedure

A flight test device (FTD), created within the IPCL as a QC tool for *Aedes* mosquitoes by measuring their flight ability [16], was adapted to compensate for the slightly larger body size of *An. arabiensis* males. The modified FTD consists of 22 transparent acrylic plastic (Polymethyl methacrylate—PMAA) tubes which are in turn housed within a larger PMAA tube. Any remaining gaps between the tubes were filled with silicon. A final larger PMAA tube which creates a containment area after the males exit the flight tubes surrounds the inner two series of tubes (all dimensions remain as described by Culbert et al. [16] except for the individual flight tube internal diameter which increased from 8 to 10 mm). The flight ability test was conducted by mouth aspirating a sample of 100 male mosquitoes into the entry hole at the base of the FTD, with 2 repetitions for the control and each experimental group. Adults were allowed a period of 2 h to escape following which, the number of mosquitoes remaining at the base of the FTD and those that had escaped were counted. Flight ability is determined by dividing the number of adults that had escaped the flight tubes by the total number that began the experiment.

### Further assessment of the flight test device: effect of light and time to pupation on the flight ability

It is known that wild *Anopheles* males are flight active mainly at the onset of dusk, especially around time of sunset for swarming/mating and/or sugar meal [21, 22]. To integrate the impact of daily activity pattern of *Anopheles* mosquitoes on the flight ability test, the flight ability of *An. arabiensis* was evaluated in two simulated light conditions: day light (390.2 Lux) and dusk (3.9 Lux). Mosquitoes were submitted to dusk conditions 30 min prior to the tests. For each light condition, flight tests were performed on 2 groups of mosquitoes separately: (1) Mosquitoes were chilled in a climatic chamber at 4  °C for 2 h and then kept under insectary conditions (27 ± 1 °C, 70 ± 10% RH) for 90 min prior to the test. Mosquitoes were from the first pupa collection day (day 7 from seeding the eggs). (2) Mosquitoes were irradiated at pupal stage (24 ± 2 h old) at 90 Gy and kept under insectary conditions (27 ± 1 °C, 70 ± 10% RH) until tests were performed. Mosquitoes were from the second pupae collection day (day 8). A control group (not chilled and not irradiated) was used in each test. Tests were performed between 9 and 12 a.m. for the day light conditions and between 4 and 7 p.m. for the dusk conditions. Mosquitoes were kept for 30 min under dusk conditions prior to the flight tests. Three replicates were performed for all treatments (control, chilled, irradiated) in each light condition. Only the flight ability was measured. To assess the effect of time to pupation on the flight ability, data from the control groups (pupae collected on day 7 and those on day 8) were compared. Then, 6 replicates were considered for each control group. The data for control groups, chilled and irradiated groups were also compared to the results obtained for same treatments in former experiments to assess the repeatability of flight ability measures.

### Statistical analysis

To analyse the impact of the various stress treatments on day 15 survival rates, insemination rates and escape rates from the flight test device (response variables), binomial linear mixed effect models were used. Repetitions were treated as random effects and the treatment levels for irradiation, chilling and compaction were then used as fixed effects. The likelihood ratio test [23, 24] was used to ascertain the significance of the fixed effects. Binomial linear mixed effect models were also used to determine if the escape rate could be used to predict the other QC parameters (day 15 survival rates and insemination rates). This was achieved by using the QC parameters as response variables and the escape rate as a fixed effect. To describe the proportion of variance explained by the model between the observed and predicted values, the R^2^ (coefficient of determination) was then used [25, 26]. P-values and fixed-effects coefficients of all models used for data analysis can be found in Additional file 4: Tables S1–S9. To analyse the data from light intensity and time to pupation experiments, general linear mixed models were used with the treatments (chilling, irradiation, light condition, time to pupation) as fixed effects, the flight ability as response variable and replicates as random effects. The outputs can be found in Additional file 4: Tables S10–S12.

## Results

### Impact of treatments on survival and insemination rates

**Fig. 1:**
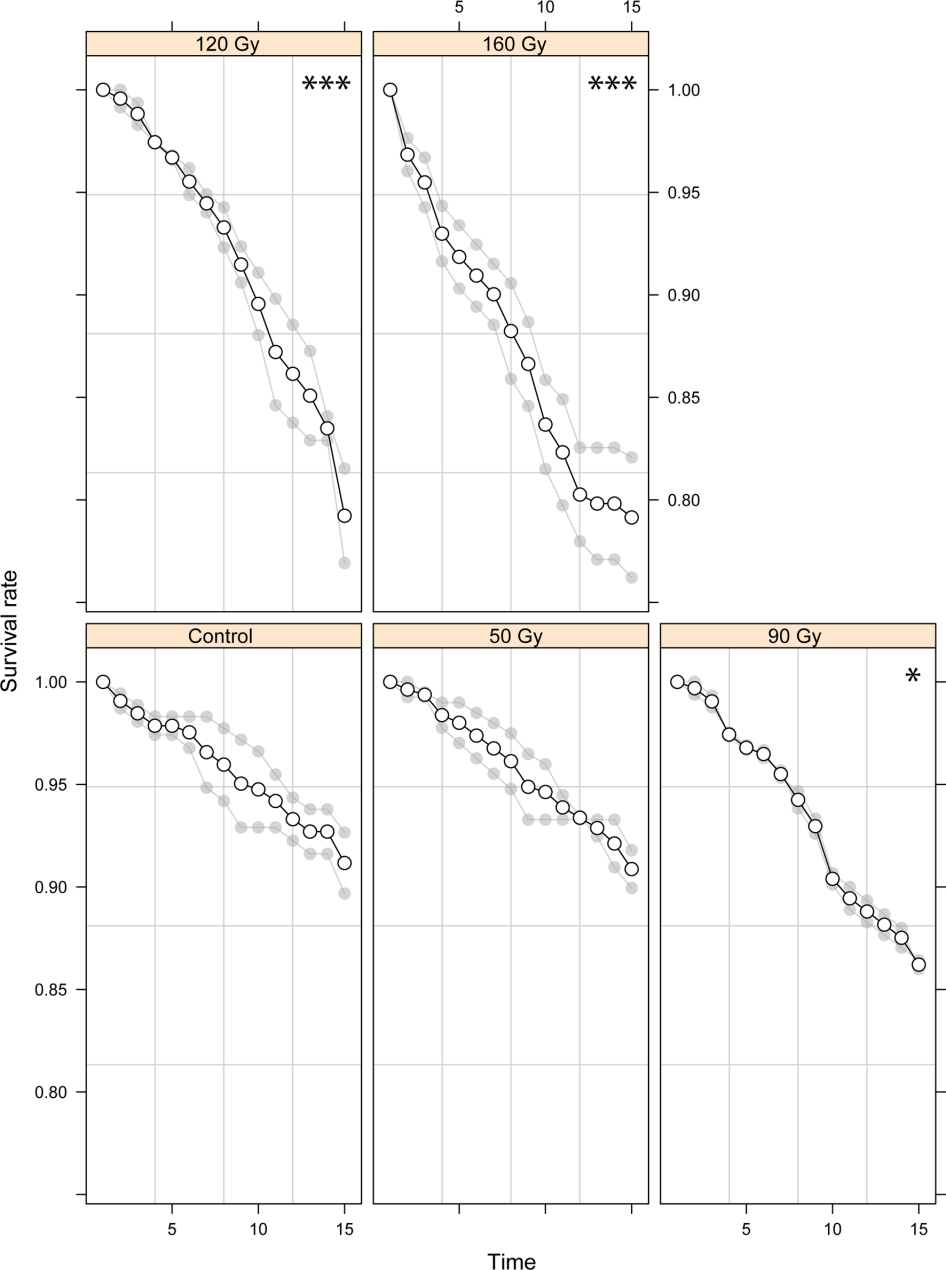
The survival rates of male *Anopheles arabiensis* subject to a range of irradiation doses for 15 days. Significant differences between the control group (no irradiation) and treatment groups (50, 90, 120 and 160 Gy) are represented as follows (*P < 0.05, **P < 0.01; ***P < 0.001). Individual values of the various replicates are indicated in light grey and mean values shown as a solid line

**Fig. 2:**
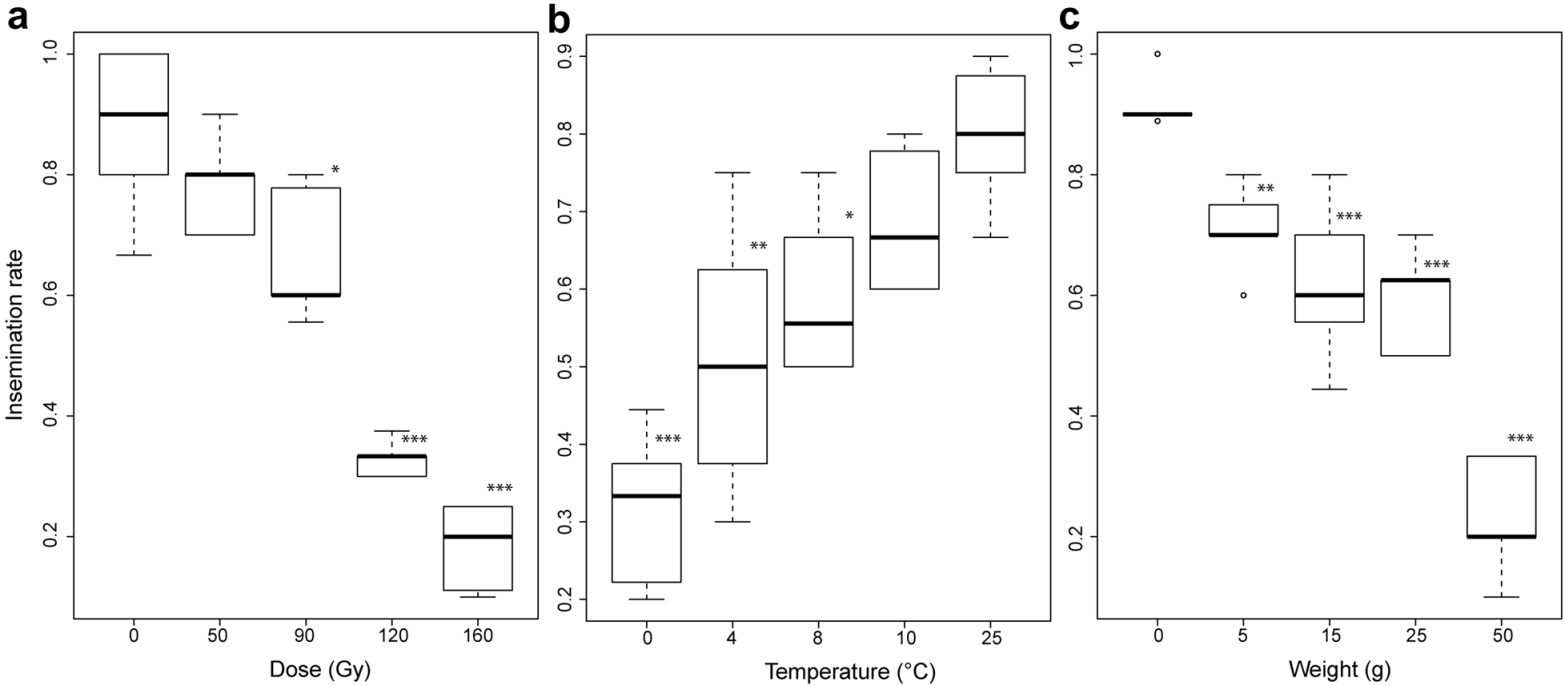
The insemination rates of male *Anopheles arabiensis* males subject to a range of stress treatments. **a** Represents the impact of various irradiation doses, **b** represents the impact of chilling temperature and **c** various compaction weights. The median value and the quartiles, horizontal bars the 95% percentiles and dots the minimal and maximal values are shown in each Boxplot with significant differences between treatment groups and the control group denoted as follows (*P < 0.05, **P < 0.01; ***P < 0.001)

The survival and insemination rate of male *Anopheles arabiensis* was significantly reduced at a dose of 90 Gy or more (Figs. 1, 2, Additional file 4: Table S1 and S4, P = 0.043, P = 0.016). Interestingly, only exposure to the lowest chilling temperature (0 °C) significantly reduced survival (Additional file 5: Fig. S1, Additional file 4: Table S2, P = 0.006), however, exposure to 8 °C or less caused a significant decrease in the insemination rate (Fig. 2, Additional file 4: Table S5, P = 0.030). A compaction weight of 15 g was found to significantly decrease survival (Additional file 6: Fig. S2, Additional file 4: Table S3, P = 0.003), whilst a weight equal to 5 g or more caused a significant impact upon the insemination rate (Fig. 2, Additional file 4: Table S6, P = 0.012).

### Impact of treatments on flight ability

**Fig. 3:**
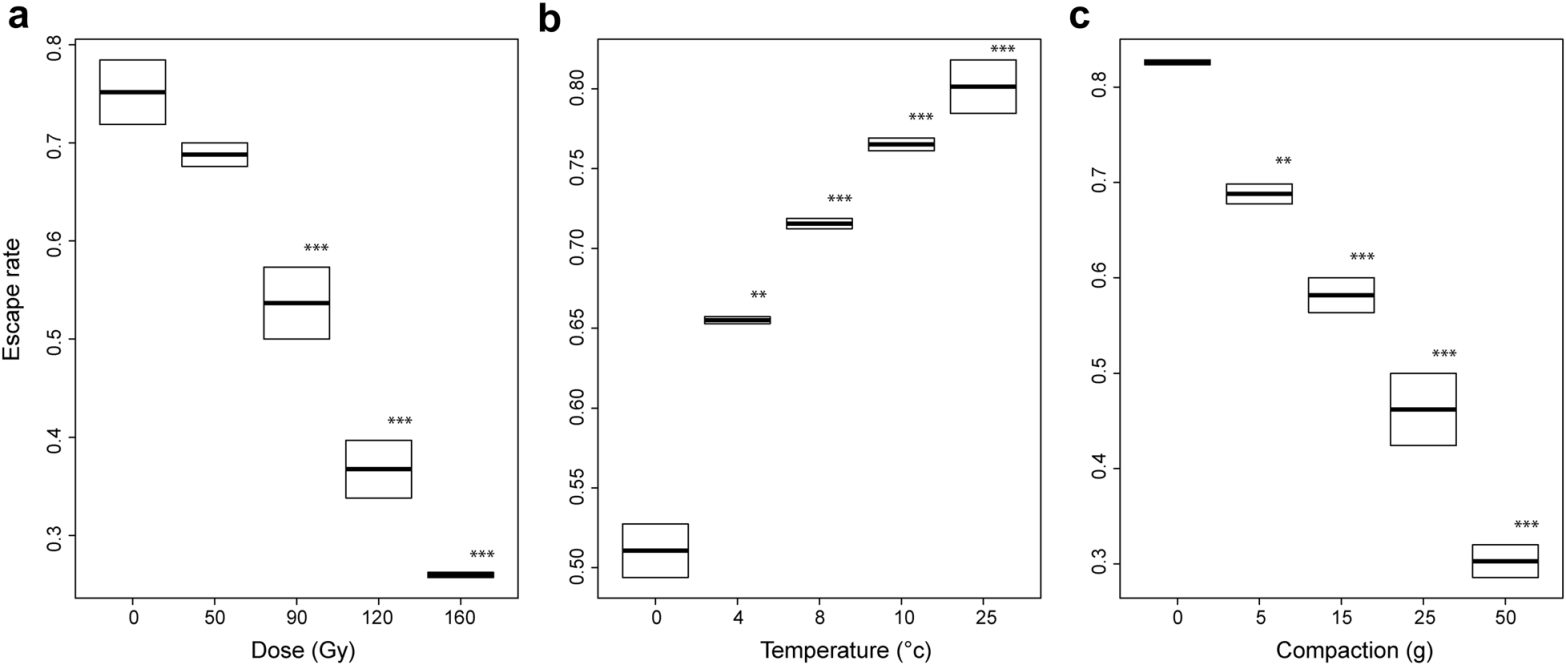
The escape rates of male *Anopheles arabiensis* mosquitoes subject to a range of stress treatments. **a** Represents the impact of various irradiation doses, **b** represents the impact of chilling temperature and **c** various compaction weights. The median value and the quartiles, horizontal bars the 95% percentiles and dots the minimal and maximal values are shown in each Boxplot with significant differences between treatment groups and the control group denoted as follows (*P < 0.05, **P < 0.01; ***P < 0.001)

**Fig. 4:**
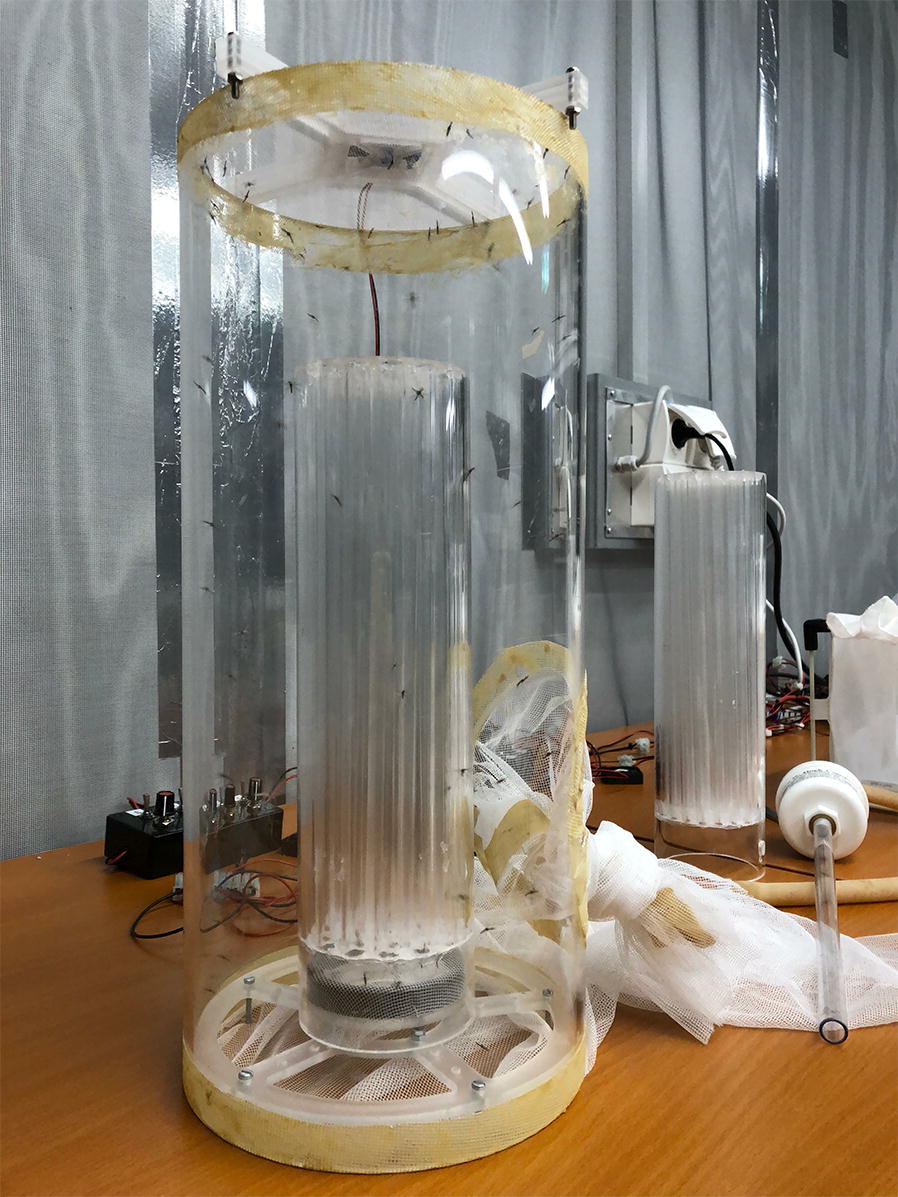
Male *Anopheles arabiensis* during a flight ability test

The results given show that the flight ability of male *An. arabiensis* is harmed by conditions of a 90 Gy dose or above, 4 °C and below and compaction of 5 g (Fig. 3, Additional file 4: Tables S7–S9, P < 0.001, P = 0.007, P = 0.009). Overall, results showed that flight ability is an accurate parameter of QC as it appeared to be sensitive to each of the various stress treatments imposed upon male *An. arabiensis* (Fig. 4, Table 1). It explained 48–98% of the variance of survival rates and 72–90% of the variance observed in insemination rates depending on the stress treatment considered. All three QC parameters significantly decreased at a dose of 90 Gy and above. Flight ability and insemination rates were most sensitive to excessive compaction (5 g). Flight ability was less sensitive to chilling (4 °C) in comparison to insemination rate (8 °C) but more sensitive than survival (0 °C).

**Table 1:** Use of the male escape rates from the flight test device to predict adult male quality parameters in *Anopheles arabiensis*

Treatment	First significant impact on escape rate	First significant impact on survival rate at day 15	First significant impact on insemination rate
Irradiation	90 Gy	90 Gy (0.75***)	90 Gy (0.90***)
Chilling	4 °C	0 °C (0.48**)	8 °C (0.72***)
Compaction	5 g	15 g (0.98***)	5 g (0.88***)

The first values of the different treatments significantly impacting each male quality indicator are presented. The values in brackets correspond to the proportion of explained variance (r-square), used as a model quality indicator, based on a linear mixed-effect model where the response variable (survival, insemination and full insemination rates) is predicted using the escape rate as a fix effect and the repeats as random effects. Survival was quantified by removing and counting dead individuals from both control and experimental cages daily for a period of 15 days. Mating propensity was calculated by measuring the number of virgin females (n = 10) a single control or post stress treatment male could successfully inseminate during a period of 4 days. Females were scored as inseminated if the spermatheca contained sperm

* 0.05 > P > 0.02, ** 0.01 > P > 0.001, *** P < 0.001

**Fig. 5:**
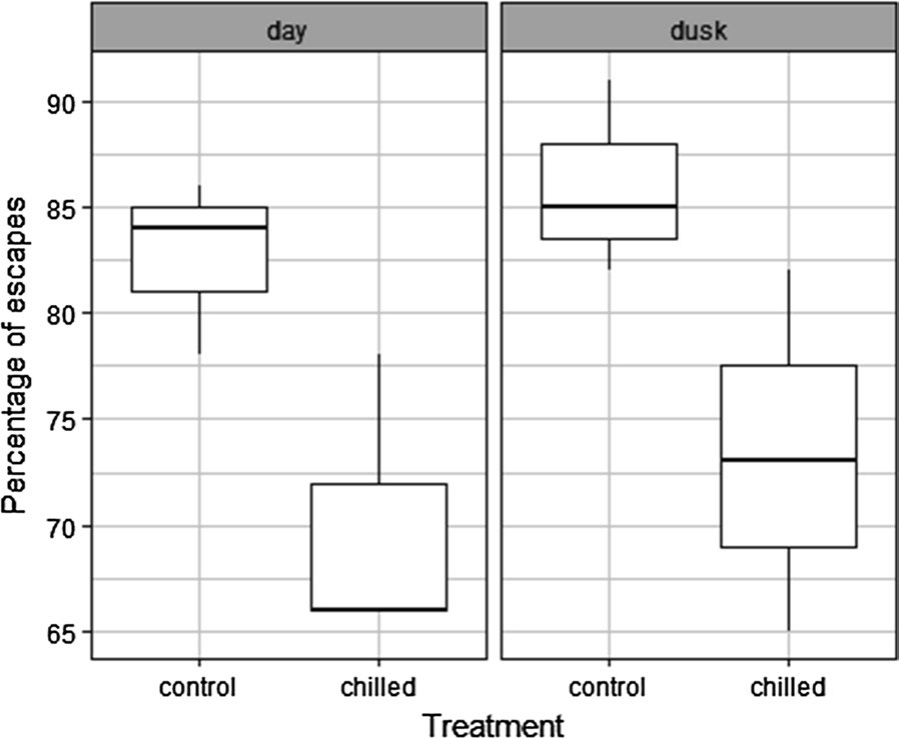
The escape rates of male *Anopheles arabiensis* mosquitoes subject to chilling at 4 °C in function of the light intensity

**Fig. 6:**
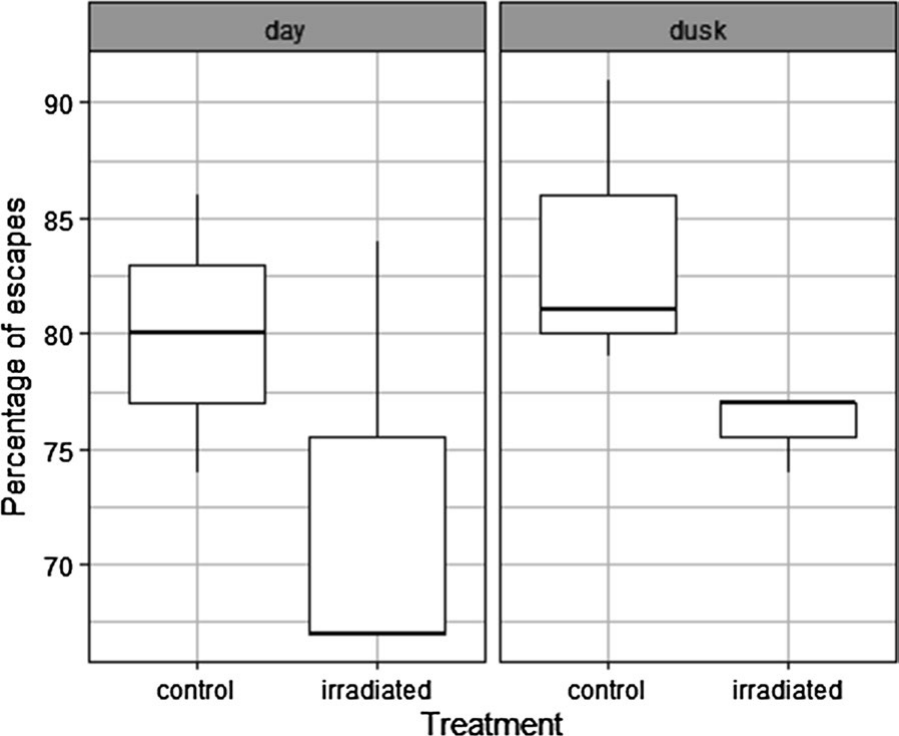
The escape rates of male *Anopheles arabiensis* mosquitoes subject to irradiation at 90 Gy in function of the light intensity

Further assessment of the flight test device confirmed that male *An. arabiensis* flight ability was significantly reduced when chilled at 4 °C for 2 h (Fig. 5, Additional file 4: Table S10, P < 0.001) or irradiated at 90 Gy (Fig. 6, Additional file 4: Table S11, P = 0.0387). However, the light did not impact the flight ability. Indeed, the best models excluded this factor. In addition, flight ability was not affected by the batch of pupa collection (first or second day of collection) (Additional file 4: Table S12, P = 0.334). Finally, the flight ability of chilled (4 °C) and control groups were compared between the first and second experiments (different operators and experiment conducted 1 year later) and no significant difference was observed (the best model did not retain the experiment number). The same result was obtained when comparing the irradiated and control groups of the two experiments. This showed a good repeatability of the flight ability tests.

## Discussion

In any release campaign, with a sterile component, it is imperative that the insects released are of adequate quality, with a maximal lifespan and minimal damage. Colonization, mass rearing, irradiation and handling are just a few examples of factors that can reduce male quality. One method of estimating insect quality is to measure their flight ability, a known reliable marker [12]. Following the development and verification of a novel flight test device to assess the quality of male *Ae. aegypti* and *Ae. albopictus* [16], a similar tool was created for male *An. arabiensis*.

The irradiation process is a crucial part of the SIT. It is critical to administer an irradiation dose that ensures a sufficient level of sterility without impacting subsequent survival and male quality, as it is well recognized that ionizing radiation can significantly decrease subsequent field competitiveness [27]. A dose of 90 Gy or more caused a significant decrease in flight ability, survival and insemination rates in *An. arabiensis* males. These results mirror those of an earlier study in which *Ae. aegypti* males exposed to 90 Gy or more displayed the same significantly reduced quality across all measured parameters [16]. It was postulated that administering a dose of 90 Gy, which falls between the partially (70 Gy) and fully sterile irradiation dose (120 Gy), to *An. arabiensis* males, would not cause a significant decrease in quality as previous studies have shown that they are still competitive even at a dose of 120 Gy [28]. As suggested previously, studies must be carried out in which male competitiveness is assessed either in semi-field or field conditions and directly compared to flight ability. It may be that flight ability offers a more accurate depiction of male mosquito quality than competitiveness measured in semi-field conditions and warrants further investigation.

Sterile insects are routinely released in a chilled state after being immobilized to facilitate the ease of their handling during their packing and transportation from the rearing facility to the release site. It seems logical to assume that a release campaign involving mosquitoes will involve a similar process as to what is currently used in other insects including Mediterranean fruit flies [14] and tsetse flies [13] and thus understanding how chilling temperature impacts male quality is fundamental to a successful campaign. A previously reported study conducted within the IPCL laboratory [10], exposed male *An. arabiensis* to a range of chilling temperatures for various durations and concluded that only exposure to 2 °C, the coldest temperature tested, for 24 h, the longest duration tested, significantly reduced survival in comparison to non-chilled control males. Therefore, it was suspected that only the lowest chilling temperature of 0 °C would significantly reduce survival. This was indeed found to be the case and, additionally, it reflects the findings of the initial verification experiments of the FTD created for *Ae. aegypti* males [16]. Despite the lesser effect of chilling temperature on male survival, regardless of species, flight ability and insemination rate are more sensitive. Significant decreases in insemination rate are apparent at 8 °C in both *An. arabiensis* and *Ae. aegypti*, whilst flight ability begins to decline at 4 and 8 °C, respectively. The impact of chilling temperature upon male quality does appear to be species-specific and thus what seems appropriate for one should not automatically be inferred for another.

The absence of impact of light on flight ability may be attributed to the attractive effect of the BG lure (Biogents, Regensburg, Germany) used in the tests. In addition, this might be explained by the adaptation of the strain to the laboratory conditions where males do not need to join swarms to mate. The result indicates that the flight ability test can be performed at any time of the day and also show a good repeatability. Moreover, the similarity of males from the first and second days of pupa collection indicates that these groups of males might be similar in fitness and then both may be considered for releases in SIT programmes.

To ensure the cost-effectiveness of a mosquito SIT programme, it is necessary to determine the maximum number of mosquitoes that can be transported in any given container without impacting their quality, or in other words the maximum tolerable level of compaction. The lowest compaction weight (5 g) was found to significantly decrease both flight ability and insemination rate. This is consistent with an earlier study conducted within the IPCL laboratory where male *An. arabiensis,* immobilized under high and low levels of compaction, did not differ in survival but did demonstrate a significantly reduced survival compared to controls under no compaction [10]. It also echoes results in the initial verification studies of the FTD using male *Ae. aegypti* where flight ability was also found to be significantly reduced following compaction in excess of 5 g [16]. Interestingly, *An. arabiensis* survival only began to significantly decrease following 15 g of compaction. Although males may be surviving after compaction under this weight, their quality was reduced as shown by their inability to fly and thus inseminate females, as is reflected in the significantly lower survival and insemination rate observed after 5 g of compaction. This finding is in keeping with a study recently published wherein several quantities (10, 40 and 240) of male *Ae. aegypti* were compacted into volumes of 1 cm^3^ and shipped across the USA [29]. Results indicated that survival was highest following the greatest level of compaction (240/cm^3^) however, a higher degree of damage was observed on the adults post-shipping. Similar outcomes from both studies highlights that not only do male mosquitoes need to survive the various stressors they encounter until their ultimate release, they need to be of an adequate quality. Survival is fruitless if they are damaged and in turn are unable to fly and mate with wild females, further facilitating the need for urgent QC tools such as the FTD.

The results of this series of experiments to validate a novel QC tool adds further support for the original version reported last year [16]. Once again, a strong correlation between flight ability and overall male quality was noted. The FTD offers a rapid and easy way of gaining insight into the quality of sterile male mosquitoes and is currently the only known tool available with such capabilities. All technical drawings of the FTD for *An. arabiensis* and also *Ae. aegypti* and *albopictus,* to allow accurate reproduction of the device, are available on website (http://www-naweb.iaea.org/nafa/ipc/public/manuals-ipc.html).

## Conclusion

In this study, results are reported from a series of verification experiments to validate a novel tool to enable the quality of *An. arabiensis* to be gauged, following minor adaptations from the original version created for *Aedes* mosquitoes. Adult males were subjected to varying levels of three different stress treatments (irradiation, chilling temperature and compaction weight) before conducting flight ability tests in the FTD. Survival and insemination rate were measured as standard reference tests and compared to flight ability to ascertain if there was a correlation. As seen in the original FTD, a new version modified for *An. arabiensis,* can accurately predict male quality and thus adds a valuable tool to the SIT toolbox. Further research could be performed to determine its suitability for use in other genetic control programmes.

## Supplementary information

**Additional file 1.** Compaction experiments raw data.

**Additional file 2.** Irradiation experiments raw data.

**Additional file 3.** Temperature experiments raw data.

**Additional file 4.** Additional tables.

**Additional file 5: Fig. S1.** The survival rates of male *Anopheles arabiensis* subject to various chilling temperatures for a period of 15 days. Significant differences between the control group (no chilling—25 °C) and treatment groups (10, 8, 4 and 0 °C) are represented as follows (*P < 0.05, **P < 0.01; ***P < 0.001). Individual values of the various replicates are indicated in light grey and mean values shown as a solid line.

**Additional file 6: Fig. S2.** The survival rates of male *Anopheles arabiensis* exposed to a variety of compaction weights for a 15 day period. Significant differences between the control group (no compaction) and treatment groups (5, 15, 25 and 50 g) are represented as follows (*P < 0.05, **P < 0.01; ***P < 0.001). Individual values of the various replicates are indicated in light grey and mean values shown as a solid line.

## Data Availability

The dataset supporting the conclusions of this article are included within the article (and its Additional files 1, 2, 3, 4).

## Abbreviations

FAO: Food and Agricultural Organization
IAEA: International Atomic Energy Agency
IPCL: Insect Pest Control Laboratory
LD: light:dark
QC: quality control
RH: relative humidity
SIT: sterile insect technique
WHO: World Health Organization
