# Supplementary material for: A rapid quality control test to foster the development of the sterile insect technique against Anopheles arabiensis
Source: Malar J. 2020 Jan 23;19:44. doi: 10.1186/s12936-020-3125-z (PMC6979282; doi:10.1186/s12936-020-3125-z)
Supplement: Supplementary file 4 — Additional file 4. Additional tables. [file 12936_2020_3125_MOESM4_ESM.docx]

Table S1. Fixed-effects coefficients of a mixed-effect binomial model of the impact

of irradiation dose on survival in *Anopheles arabiensis*

| Fixed effects | Value | Std. Error | z-value | p-value |
| --- | --- | --- | --- | --- |
| Intercept | 2.3464 | 0.1944 | 12.071 | 2e-16 |
| 50 Gy | -0.0700 | 0.2708 | -0.258 | 0.7961 |
| 90 Gy | -0.5129 | 0.2545 | -2.016 | 0.0438 |
| 120 Gy | -0.9873 | 0.2454 | -4.023 | 5.75e-05 |
| 160 Gy | -1.0189 | 0.2270 | -4.488 | 7.18e-06 |

Table S2. Fixed-effects coefficients of a mixed-effect binomial model of the impact of chilling temperature on survival in *Anopheles arabiensis.*

| Fixed effects | Value | Std. Error | z-value | p-value |
| --- | --- | --- | --- | --- |
| Intercept | 1.1872 | 0.1555 | 7.636 | 2.23e-14 |
| 0 °C | -0.5672 | 0.2056 | -2.759 | 0.00579 |
| 4 °C | -0.2633 | 0.2046 | -1.287 | 0.19806 |
| 8 °C | -0.1296 | 0.2177 | -0.595 | 0.55161 |
| 10 °C | -0.1477 | 0.2180 | -0.678 | 0.49788 |

Table S3. Fixed-effects coefficients of a mixed-effect binomial model of the impact

of compaction on survival in *Anopheles arabiensis*

| Fixed effects | Value | Std. Error | z-value | p-value |
| --- | --- | --- | --- | --- |
| Intercept | 2.2357 | 0.2552 | 8.762 | 2e-16 |
| 5 g | -0.5216 | 0.3253 | -1.603 | 0.10883 |
| 15 g | -0.8833 | 0.3051 | -2.895 | 0.00379 |
| 25 g | -1.3194 | 0.2974 | -4.436 | 9.14e-06 |
| 50 g | -2.1922 | 0.2947 | -7.438 | 1.02e-13 |

Table S4. Fixed-effects coefficients of a mixed-effect binomial model of the impact

of irradiation dose on insemination rate in *Anopheles arabiensis*

| Fixed effects | Value | Std. Error | z-value | p-value |
| --- | --- | --- | --- | --- |
| Intercept | 1.9694 | 0.4358 | 4.519 | 6.21e-06 |
| 50 Gy | -0.7038 | 0.5536 | -1.271 | 0.2036 |
| 90 Gy | -1.2763 | 0.5326 | -2.396 | 0.0166 |
| 120 Gy | -2.6954 | 0.5374 | -5.015 | 5.30e-07 |
| 160 Gy | -3.5009 | 0.5848 | -5.987 | 2.14e-09 |

Table S5. Fixed-effects coefficients of a mixed-effect binomial model of the impact

of chilling temperature on insemination rate in *Anopheles arabiensis*

| Fixed effects | Value | Std. Error | z-value | p-value |
| --- | --- | --- | --- | --- |
| Intercept | 1.3863 | 0.3727 | 3.720 | 0.000199 |
| 0 °C | -2.1812 | 0.4925 | -4.429 | 9.48e-06 |
| 4 °C | -1.3863 | 0.4794 | -2.892 | 0.003829 |
| 8 °C | -1.0349 | 0.4781 | -2.165 | 0.030410 |
| 10 °C | -0.5978 | 0.4857 | -1.231 | 0.218324 |

Table S6. Fixed-effects coefficients of a mixed-effect binomial model of the impact

of compaction on insemination rate in *Anopheles arabiensis*

| Fixed effects | Value | Std. Error | z-value | p-value |
| --- | --- | --- | --- | --- |
| Intercept | 2.4204 | 0.5217 | 4.639 | 3.50e-06 |
| 5 g | -1.5331 | 0.6108 | -2.510 | 0.012074 |
| 15 g | -1.9095 | 0.6009 | -3.178 | 0.001485 |
| 25 g | -2.0690 | 0.6016 | -3.439 | 0.000583 |
| 50 g | -3.6334 | 0.6246 | -5.817 | 5.99e-09 |

Table. S7. Fixed-effects coefficients of a mixed-effect binomial model of the impact

of irradiation dose on the escape rate from the flight organ in *Anopheles arabiensis*

| Fixed effects | Value | Std. Error | z-value | p-value |
| --- | --- | --- | --- | --- |
| Intercept | 1.1090 | 0.2039 | 5.440 | 5.33e-08 |
| 50 Gy | -0.3185 | 0.2731 | -1.166 | 0.243625 |
| 90 Gy | -0.9526 | 0.2648 | -3,598 | 0.000321 |
| 120 Gy | -1.6513 | 0.2705 | -6.104 | 1.04e-09 |
| 160 Gy | -2.1549 | 0.2761 | -7.806 | 5.90e-15 |

Table. S8. Fixed-effects coefficients of a mixed-effect binomial model of the impact of chilling temperature on the escape rate from the flight organ in *Anopheles arabiensis*

| Fixed effects | Value | Std. Error | z-value | p-value |
| --- | --- | --- | --- | --- |
| Intercept | 1.3959 | 0.2191 | 6.372 | 1.86e-10 |
| 0 °C | -1.3493 | 0.2669 | -0.5055 | 4.30e-07 |
| 4 °C | -0.7551 | 0.2813 | -2.684 | 0.00727 |
| 8 °C | -0.4745 | 0.2895 | -1.639 | 0.10128 |
| 10 °C | -0.2147 | 0.3002 | -0.715 | 0.47449 |

Table. S9. Fixed-effects coefficients of a mixed-effect binomial model of the impact

of compaction on the escape rate from the flight organ in *Anopheles arabiensis*

| Fixed effects | Value | Std. Error | z-value | p-value |
| --- | --- | --- | --- | --- |
| Intercept | 1.5581 | 0.2246 | 6.938 | 3.98e-12 |
| 5 g | -0.7674 | 0.2962 | -2.591 | 0.00957 |
| 15 g | -1.2264 | 0.2804 | -4.374 | 1.22e-05 |
| 25 g | -1.7012 | 0.2813 | -6.047 | 1.48e-09 |
| 50 g | -2.3911 | 0.3135 | -7.628 | 2.39e-14 |

Table S10. Effect of chilling on male *Anopheles arabiensis* flight ability

|  |  | Estimate | Standard error | z value | Pr(>\|z\|) |
| --- | --- | --- | --- | --- | --- |
| (Intercept) | | 1.6418 | 0.1279 | 12.839 | <2e-16 *** |
| Chilled mosquitoes | | -0.7255 | 0.1647 | -4.405 | 1.06e-05 *** |

Reference level for regression: Control (Not chilled mosquitoes)

Significance codes : 0 ‘***’ 0.001 ‘**’ 0.01 ‘*’ 0.05

The best model excluded the light condition which did not affect the flight ability

Table S11. Effect of irradiation on male *Anopheles arabiensis* flight ability

|  |  | Estimate | Standard error | z value | Pr(>\|z\|) |
| --- | --- | --- | --- | --- | --- |
| (Intercept) | | 1.4617 | 0.1356 | 10.780 | <2e-16 *** |
| Irradiated mosquitoes | | -0.4025 | 0.1947 | -2.068 | 0.0387 * |

Reference level for regression: Control (Not irradiated mosquitoes)

Significance codes : 0 ‘***’ 0.001 ‘**’ 0.01 ‘*’ 0.05

The best model excluded the light condition which did not affect the flight ability

Table S12. Effect of time to pupation on male *Anopheles arabiensis* flight ability

|  |  | Estimate | Standard error | z value | Pr(>\|z\|) |
| --- | --- | --- | --- | --- | --- |
| (Intercept) | | 1.6418 | 0.1279 | 12.839 | <2e-16 *** |
| Second day of pupae collection | | -0.1801 | 0.1864 | -0.966 | 0.334 |

Reference level for regression: First day of pupa collection (Not irradiated mosquitoes)

Significance codes : 0 ‘***’ 0.001 ‘**’ 0.01 ‘*’ 0.05
