# Supplementary figures and images for: A rapid quality control test to foster the development of the sterile insect technique against Anopheles arabiensis
Source: Malar J. 2020 Jan 23;19:44. doi: 10.1186/s12936-020-3125-z (PMC6979282; doi:10.1186/s12936-020-3125-z)

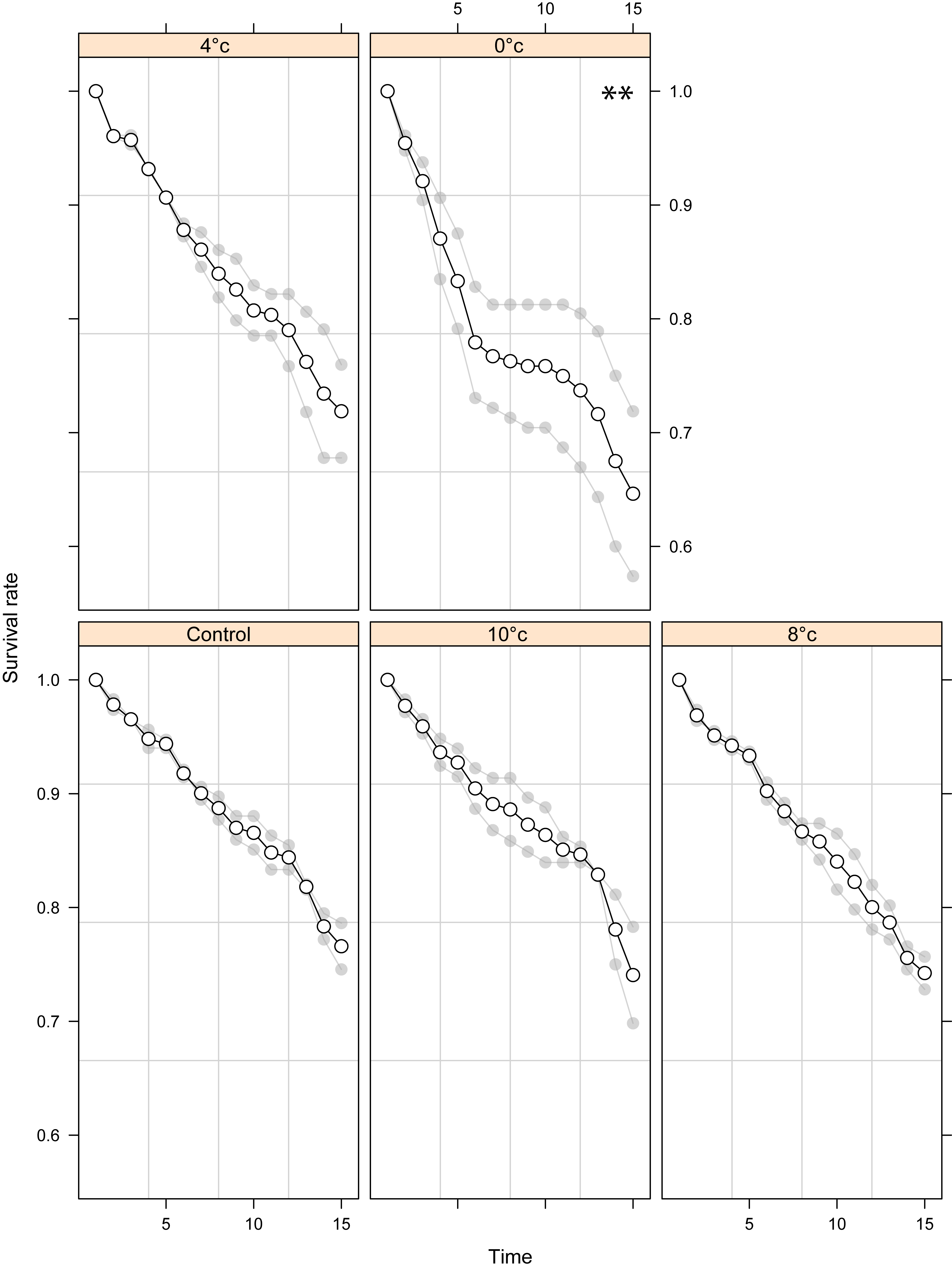

Supplement: Supplementary file 5 — Additional file 5: Fig. S1. The survival rates of male Anopheles arabiensis subject to various chilling temperatures for a period of 15 days. Significant differences between the control group (no chilling—25 °C) and treatment groups (10, 8, 4 and 0 °C) are represented as follows (*P < 0.05, **P < 0.01; ***P < 0.001). Individual values of the various replicates are indicated in light grey and mean values shown as a solid line. [file 12936_2020_3125_MOESM5_ESM.tif]

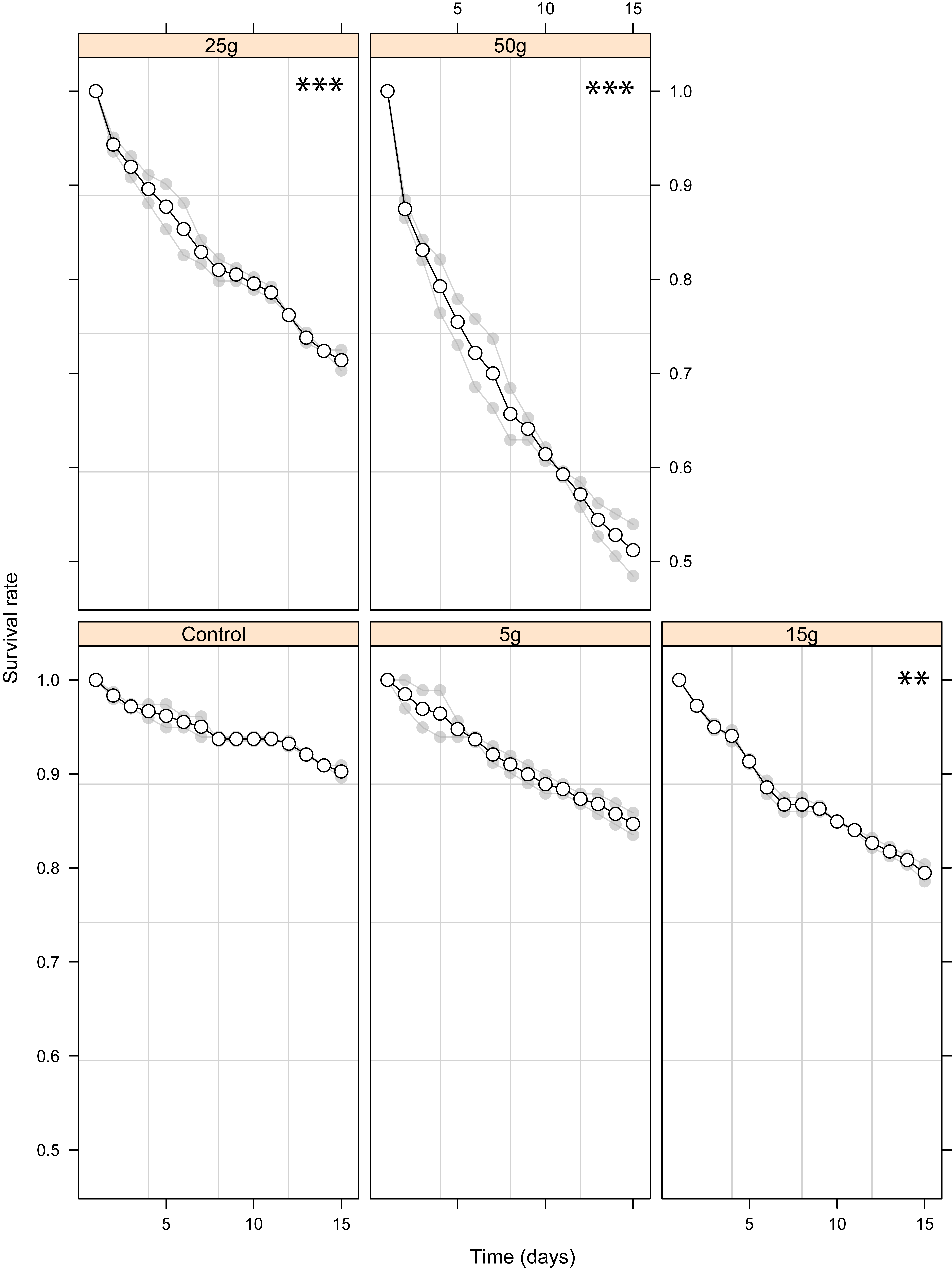

Supplement: Supplementary file 6 — Additional file 6: Fig. S2. The survival rates of male Anopheles arabiensis exposed to a variety of compaction weights for a 15 day period. Significant differences between the control group (no compaction) and treatment groups (5, 15, 25 and 50 g) are represented as follows (*P < 0.05, **P < 0.01; ***P < 0.001). Individual values of the various replicates are indicated in light grey and mean values shown as a solid line. [file 12936_2020_3125_MOESM6_ESM.tif]
